# Plant Salinity Stress: Many Unanswered Questions Remain

**DOI:** 10.3389/fpls.2019.00080

**Published:** 2019-02-15

**Authors:** Stanislav V. Isayenkov, Frans J. M. Maathuis

**Affiliations:** ^1^ Department of Plant Food Products and Biofortification, Institute of Food Biotechnology and Genomics NAS of Ukraine, Kyiv, Ukraine; ^2^ Department of Biology, University of York, York, United Kingdom

**Keywords:** salt stress, role of K^+^, transport of Na^+^ and Cl^−^, mechanisms of salt tolerance, membrane transporters, ion uptake, symplastic and apoplastic pathway

## Abstract

Salinity is a major threat to modern agriculture causing inhibition and impairment of crop growth and development. Here, we not only review recent advances in salinity stress research in plants but also revisit some basic perennial questions that still remain unanswered. In this review, we analyze the physiological, biochemical, and molecular aspects of Na^+^ and Cl^−^ uptake, sequestration, and transport associated with salinity. We discuss the role and importance of symplastic versus apoplastic pathways for ion uptake and critically evaluate the role of different types of membrane transporters in Na^+^ and Cl^−^ uptake and intercellular and intracellular ion distribution. Our incomplete knowledge regarding possible mechanisms of salinity sensing by plants is evaluated. Furthermore, a critical evaluation of the mechanisms of ion toxicity leads us to believe that, in contrast to currently held ideas, toxicity only plays a minor role in the cytosol and may be more prevalent in the vacuole. Lastly, the multiple roles of K^+^ in plant salinity stress are discussed.

## General Aspects of Plant Salt Stress

Soil salinity is one of the most important global problems that negatively affects crop productivity. Salinity impairs plant growth and development *via* water stress, cytotoxicity due to excessive uptake of ions such as sodium (Na^+^) and chloride (Cl^−^), and nutritional imbalance. Additionally, salinity is typically accompanied by oxidative stress due to generation of reactive oxygen species (ROS) (Tsugane et al., 1999; Hernandez et al., 2001; Isayenkov, 2012).

Plant responses to salinity have been divided into two main phases. An ion-independent growth reduction, which takes place within minutes to days, causes stomatal closure and inhibition of cell expansion mainly in the shoot (Munns and Passioura, 1984; Munns and Termaat, 1986; Rajendran et al., 2009). A second phase takes place over days or even weeks and pertains to the build-up of cytotoxic ion levels, which slows down metabolic processes, causes premature senescence, and ultimately cell death (Munns and Tester, 2008; Roy et al., 2014). Tolerance to both types of stress is governed by a multitude of physiological and molecular mechanisms: osmotic tolerance, ionic tolerance, and tissue tolerance (Rajendran et al., 2009; Roy et al., 2014). Osmotic tolerance initiates relatively quickly and includes a rapid decrease in stomatal conductance to preserve water. It employs fast long-distance (root to shoot) signaling mechanisms (Ismail et al., 2007; Maischak et al., 2010; Roy et al., 2014), which largely do not discriminate between osmotic effects created by NaCl, KCl, mannitol, or polyethylene glycol (Yeo et al., 1991; Chazen et al., 1995).

The entering of salt into the root system triggers activation of several signal cascades that generate ionic tolerance by restricting (net) Na^+^ influx into the root and reduce (net) Na^+^ translocation. Lastly, tissue tolerance is enhanced by compartmentation of toxic ions into vacuoles to avoid detrimental effects on cytoplasmic processes. The above strategies have been observed in many types of plant, and differences in tolerance between glycophytes and halophytes are predominantly due to the greater robustness of the employed mechanisms in the latter, rather than a qualitative difference (Flowers and Colmer, 2008, 2015; Maathuis et al., 2014). Most of these aspects have been covered in previous reviews; here, we will focus particularly on the quantitative role of symplastic and the apoplastic pathways regarding salt influx, an evaluation of how mechanisms of chloride uptake, transport, and distribution, compare to that of sodium and a critical re-evaluation of ion toxicity.

## How does Salt Enter the Plant?

Salinity creates a dilemma for plants; increased levels of inorganic minerals in the environment create osmotic and water stress but at the same time provide cheap osmoticum to lower the cell osmotic potential and hence prevent water loss. In spite of decades of research, one of the most enigmatic questions relating to plant salt stress remains the mechanism(s) by which Na^+^ and Cl^−^ enter roots.

**Figure 1:**
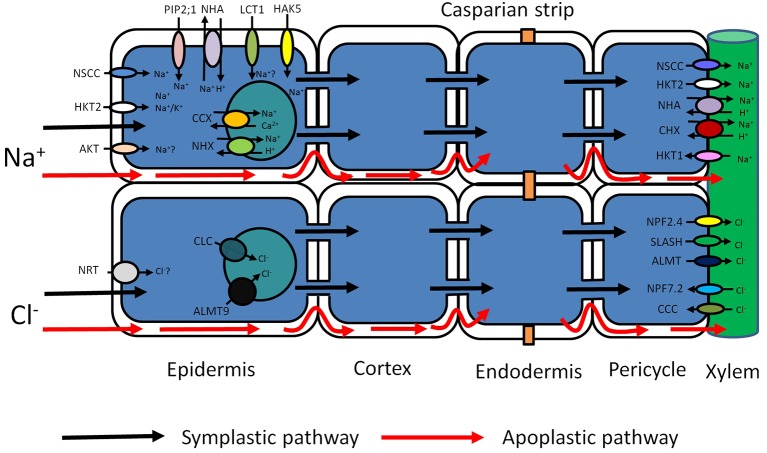
Schematic representation of possible transport pathways for Na^+^ and Cl^−^ uptake and their cellular and long-distance distribution. Red arrows represent Na^+^ and Cl^−^ entry sites and route through cell walls – apoplastic bypass flow. Black arrows represent Na^+^ and Cl^−^ entry sites and cytoplasmic route through plasma membrane-symplastic pathway. Various transporters (AKT, HKT2, NSCC, PIP2;1, NHA, LCT1, HAK5) may be involved in Na^+^ uptake and movement through the plasma membrane. Compartmentalization of Na+ in vacuoles is mediated by tonoplast transporters (CCX, NHX). The further Na^+^ redistribution over long distances may rely on members of several membrane transporter families (NSCC, HKT, NHA, CHX). Cl^−^ entry to the root cells through the plasma membrane may be mediated by Cl^−^/H^+^ co-transporter NRT. Vacuolar Cl^−^ sequestration may possibly be performed by two anion tonoplast transporters (ALMT and CLC). Cl^−^ membrane transport over long distances may be conducted by membrane transporters from different protein families (NPF, SLASH, ALMT, NPF, CCC). AKT, Arabidopsis K^+^ transporter; HKT, High-affinity K^+^ transporter Type; NSCC, Nonselective cation channels; PIP 2,1, Plasma membrane intrinsic protein (Aquaporin); NHA, Na^+^/H^+^ antiporter (SOS1); LCT1, Low-affinity cation transporter; HAK, High-affinity K^+^ uptake transporter; CHX, cation/H^+^ exchanger; NHX, Na^+^/H^+^ exchanger; NRT, Nitrate transporter; ALMT, Aluminum-activated malate transporter; CLC, Chloride channel; NPF, Nitrate transporter 1/peptide transporter; CCC, Cation/chloride cotransporter; SLASH, Anion channel associated homolog 1.

Ion uptake can occur *via* the symplastic and the apoplastic pathway (Figure 1; Gao et al., 2007; Negrão et al., 2011; Maathuis et al., 2014). The apoplastic pathway is a direct flow continuum between the outside and the xylem (Yeo et al., 1987; Anil et al., 2005; Krishnamurthy et al., 2009). In most conditions, the contribution of this “bypass” flux is less than 1% of the transpirational volume flow (Hanson et al., 1985; Moon et al., 1986; Yeo et al., 1987). Nevertheless, this can be much greater when transpirational demand is high (Pitman, 1982; Sanderson, 1983). In rice, it is particularly pronounced and could be responsible for up to 50% of total Na^+^ uptake (Yeo et al., 1987; Malagoli et al., 2008; Krishnamurthy et al., 2009; Kronzucker and Britto, 2011). Significant apoplastic Na^+^ flux has also been reported in other species (Peterson et al., 1981, 1986), and recently, it was shown that up to 50% of Cl^−^ translocation to rice shoots is also apoplastic (Shi et al., 2013). Although particularly pronounced in rice, the combined data suggest that nonsymplastic Na^+^ and Cl^−^ uptake may be very relevant in monocots. However, solute permeability coefficients in Arabidopsis roots are not that different from those in rice (Ranathunge and Schreiber, 2011), and detailed experimentation is needed to test the contribution of the apoplastic pathway in this model and other dicots (Figure 1).

Net uptake *via* the symplastic pathway of Na^+^ (Cl^−^) into roots is assumed to be catalyzed by a specific complement of transporters (Figure 1). Evidence points to a large number of different systems, but their relative contribution, and therefore physiological relevance, is often unclear. Nonselective cation channels (NSCCs) are encoded by two gene families: glutamate receptor-like channels (GLRs) and cyclic nucleotide-gated channels (CNGCs) and blocked by Ca^2+^ (Leng et al., 2002; Demidchik et al., 2004, 2018; Demidchik and Maathuis, 2007). The apoplastic Ca^2+^ concentration in root cells is probably in the region of 0.2–0.4 mM (Legué et al., 1997), which is enough to reduce NSCC-mediated flux by 30–50% (Essah et al., 2003). The remaining flux can be further diminished not only by a number of channel blockers like Gd^3+^ and La^3+^ (Demidchik and Maathuis, 2007) but also by organic compounds like cyclic GMP. Thus, in plants like Arabidopsis, it appears that a large fraction of inward Na^+^ flux is carried by NSCCs but either the genetic identity of the contributing channels is obscure or their putative role has not been quantified. For example, though AtCNGC3 channels impacted on salt-related growth (Gobert et al., 2006), whether they directly affected Na^+^ uptake was not measured.

In monocotyledonous plants, the situation is likely to be different. In contrast to Arabidopsis, which contains only the subclass 1, Na^+^ selective, AtHKT1 isoform, monocots have multiple HKT isoforms. Arabidopsis HKT1 functions in long-distance transport of Na^+^ *via* xylem and phloem (Berthomieu et al., 2003; Sunarpi et al., 2005), but in several cereals HKTs can mediate Na^+^ uptake: In rice, OsHKT2;1 catalyzes Na^+^ uptake in low K^+^, low Na^+^ (<2 mM) conditions (Horie et al., 2007). Overexpression of HvHKT2;1 in barley causes increased Na^+^ uptake in salt stress conditions (Mian et al., 2011) with a Km for Na^+^ transport in the low-affinity region (3–6 mM). Similarly, altered expression of TaHKT2;1 in wheat affected Na^+^ accumulation in the low-affinity range (Laurie et al., 2002), but detailed flux studies are lacking (Figure 1).

Intriguingly, electrophysiological experiments in *Xenopus* oocytes showed considerable Na^+^ conductance when heterologously expressing the aquaporin AtPIP2;1 from *Arabidopsis* (Byrt et al., 2017). This interesting phenomenon was PIP isoform specific. A dual ion and water conducting capability, as suggested for AtPIP2;1, could couple ion and water flux, which would have obvious physiological relevance. However, in intact roots, unidirectional Na^+^ influx is typically in the range of 20–200 μmol gFW^−1^ h^−1^ (Kronzucker and Britto, 2011). Using a conversion factor of 5 × 10^−4^ m^2^ gFW^−1^ (Ahmad et al., 2015), this equates to 0.1–1 mol m^−2^ s^−1^. In comparison, PIP2;1 generated fluxes in oocytes were in the order of 0.2–0.5 μmol m^−2^ s^−1^ (Byrt et al., 2017), ~6 orders of magnitude smaller. Thus, the extent to which aquaporins contribute to Na^+^ uptake in intact plants appears to be negligible (Figure 1).

The low-affinity cation transporter LCT1 (Schachtman et al., 1997; Clemens et al., 1998; Kronzucker and Britto, 2011), when expressed in yeast, functions as a nonselective cation carrier and is capable of transporting K^+^, Rb^+^, Na^+^, and Ca^2+^ (Schachtman et al., 1997; Clemens et al., 1998). Moreover, its expression in yeast resulted in increased salt sensitivity (Amtmann et al., 2001), promoting the hypothesis that LCT1 could mediate Na^+^ uptake in planta. Nevertheless, more recent analyses suggest that LCT1 is not directly involved in Na^+^ transport (Figure 1; Plett and Moller, 2010).

A combination of high salinity and low K^+^ could cause Na/K ratios over 10^3^ fold, a value that exceeds the K/Na selectivity of many K^+^ channels (Maathuis, 2014). Shaker type K^+^ channels, such as KAT1 and AKT1, are involved in K^+^ uptake and, on the basis of detailed electrophysiological studies, were deemed not to participate in Na^+^ transport (Schachtman et al., 1991; Amtmann and Sanders, 1998). However, later studies found that in intact tissue the Na-influx inhibitor profile mostly aligned with that of AKT1 type channels in the halophytic plant *Suaeda maritima* (Wang et al., 2007) and in rice (Kader and Lindbergh, 2005). Data from the halophyte study showed 30–40% reduction in unidirectional Na^+^ influx in the presence of “classical” K^+^ channel blockers such as Cs, Ba^2+^, and TEA, suggesting that up to 30–40% of Na^+^ influx could occur *via* AKT1 type channels. However, these types of studies are notoriously difficult to interpret because most blockers show only limited selectivity. Furthermore, flux assays did not show any lower Na^+^ influx in *akt*1 KO mutants compared to WT Arabidopsis (Figure 1; Essah et al., 2003).

In contrast to Na^+^, Cl^−^ is an essential nutrient for plants. It has been postulated that Cl^−^ is transported into the cell by a H^+^/Cl^−^ symport, but its molecular nature is unknown. When plants are exposed to salinity, the external [Cl^−^] may be sufficiently high for a fraction of Cl^−^ to enter passively through anion channels, but the relevant transport mechanism is this case too is unknown. Another class of potential Cl^−^ transporters is the cation chloride cotransporters (CCCs), a notion that has so far received little attention but is supported by the observation that 100 μM bumetanide drastically reduces Na^+^ uptake in *Suaeda maritima* (Zhang et al., 2011). In Arabidopsis, AtCCC has been studied in some detail, showing that it is expressed in root and shoot tissues and most likely involved in coordinated K^+^, Na^+^, and Cl^−^ symport (Colmenero-Flores et al., 2007; Zhang et al., 2010). However, loss of function in AtCCC led to an increase in Cl^−^ uptake (Colmenero-Flores et al., 2007), arguing against a role of AtCCC in Cl^−^ uptake (Figure 1). This study indicates that CCC transporters from grapevine and Arabidopsis are targeted to the Golgi and Trans-Golgi network and indirectly influence long-distance ion transport and plant salt tolerance (Henderson et al., 2015). According to these data, the AtCCC like VvCCC is involved in the transport of Na^+^, K^+^, Cl^−^ and contributes to Na^+^ and Cl^−^ homeostasis (Henderson et al., 2015).

In summary, for the majority of species and conditions, there is considerable evidence suggesting that NSCCs are a main pathway for Na^+^ influx into roots in glycophytic plants but these may subsume multiple channels from multiple channel families. This complexity impedes the construction of a detailed picture regarding “who does what” and by how much. In monocots, members of the HKT family could contribute to Na^+^ uptake. The usage of mutants should allow us to make progress regarding Cl^−^ uptake. Existing data show that for aquatic plants like rice with its specific root anatomy, the apoplastic bypass of Na^+^ and Cl^−^ can be considerable.

## Early Components of Salinity Sensing in Plants

The plant response to salinity is complex but presumably includes some mechanism to report increasing levels of ions, either in the external medium or within the symplast. However, how Na^+^ or Cl^−^ is sensed by plants remains unknown. In animal systems, primary Na^+^ sensors typically rely on functioning of specific Na^+^ selective ion channels with Na^+^ binding sites that modulate the gate and thus are capable of functioning as reporters of body fluid Na^+^ levels. In other cell types such as taste buds, Enac-type Na^+^ channels cause depolarizations proportionate to the amount of Na^+^ that is present (Maathuis, 2014) while sensory cells of nematode cilia have “transmembrane channel like” (TMC) channels activated by Na^+^ concentration higher than 140 mM causing an avoidance reaction (Chatzigeorgiou et al., 2013).

**Figure 2:**
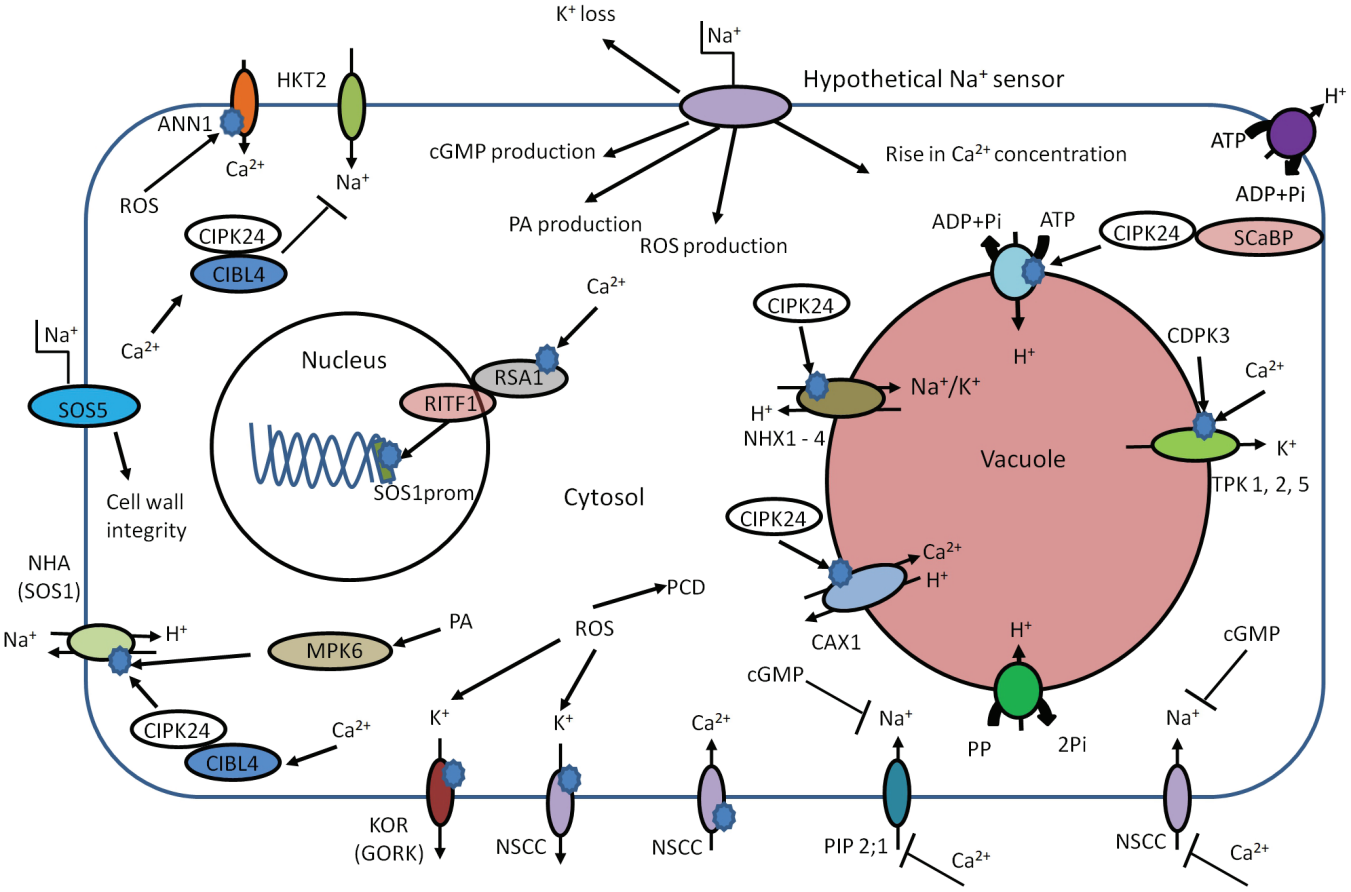
Schematic overview of early components involved in salt sensing. High external Na^+^ concentration leads to elevation of intracellular Ca^2+^, phosphatidic acid (PA), and cGMP. Cld PA can activate NHA (SOS1) in an independent manner. The main target of Ca^2+^ is CIBL4 (SOS3). The CIBL4 is capable to form the complex with CBL-interacting serine/threonine-protein kinase 24 (CIPK24, SOS2). The CIBL4-CIPK24 complex activates NHA (SOS1) and inhibits Na^+^ uptake by HKT2. CIPK24 together with SCaBP (SOS3 like protein) is involved in activation of the V-ATPase. CIPK24 participates in the activation of vacuolar transporters such as CAX and NHX. The rise in cytosolic Ca^2+^ concentration could trigger interaction of RSA1-RITF1. RSA1-RITF1 complex activates promoter of *SOS1* gene. PA is involved in activation of mitogen-activated protein kinase 6 (MPK6). MPK6 can directly phosphorylate SOS1. The Ca^2+^-dependent kinase (CDPK3) and cytosolic Ca^2+^ lead to activation of vacuolar two-pore K^+^ channels (TPKs) and subsequent K^+^ release from vacuole. Due to the plasma membrane localization, SOS5 protein is considered to be potential candidate for extracellular Na^+^ sensing and helps maintain of cell wall integrity and architecture. Annexin1 (ANN1) is capable to sense the high concentrations of extracellular Na^+^ by mediating ROS-activated Ca^2+^ influx through the plasma membrane of plant cells. The rise of cGMP leads to inhibition of Na^+^ uptake, possibly *via* cyclic nucleotide–gated ion channels (CNGSs) and glutamate receptor (GLRs). PIP2;1, CNGCs, and GLRs could be blocked by exogenous Ca^2+^. The ROS production leads to K^+^ leak *via* activation of outward K^+^ channels – KOR (guard cells outward K^+^ channel, GORK) and NSCC. The intracellular accumulation of ROS at high levels can trigger programmed cell death (PCD).

Thus, primary sensors in animals typically rely on Na^+^ specific binding sites that modulate transporter activity. As yet, no similar mechanisms have been identified in plant species but other, rapid responses such as salt-induced membrane depolarization and Ca^2+^ signals could form early components of salt sensing relays. However, membrane depolarizations do not confer any specificity so are unlikely to be physiologically relevant. Increases in extracellular NaCl cause rapid Ca^2+^ elevation in the cytosol (Knight et al., 1997), but these are often similar to signals induced by equiosmolar levels of osmotica such as mannitol. However, in some cases, the Ca^2+^ signals are salt specific (e.g. Choi et al., 2014), although tests for ionic specificity are usually lacking. Unfortunately, the upstream components of the Ca^2+^ signal are entirely unknown. New insights such as those from Choi et al. (2016) show that long-distance Ca^2+^ waves in response to salinization might form useful tools to devise mutant screens that could point to upstream components. Reactive oxygen species (ROS) may constitute another potential component upstream of the Ca^2+^ signal: Annexin1 (AtANN1) from *Arabidopsis thaliana* responds to high extracellular NaCl by mediating ROS-activated Ca^2+^ influx through the plasma membrane of plant cells (Laohavisit et al., 2013). Thus, annexin 1 could be an early key component of root cell adaptation to salinity (Laohavisit et al., 2013). Downstream, Ca^2+^-dependent signaling can be propagated by calcium-dependent protein kinases (CDPKs) and calcineurin B-like proteins (CBLs) (Weinl and Kudla, 2008) with CBL-interacting protein kinases (CIPKs) (Boudsocq and Sheen, 2013), which in turn modulate protein activity and gene transcription (Figure 2).

One CBL (CBL4 or SOS3; salt over sensitive) is responsible for sensing calcium signals caused by salinity (Figure 2). The calcium binds to CBL4 causing dimerization of this protein and enhancing the activity of CIPK24 (SOS2) serine/threonine protein kinase. The resulting CBL4/CIPK24 (SOS3/SOS2) complex activates the Na^+^/H^+^ antiporter–SOS1 *via* phosphorylation (Zhu, 2002; Martínez-Atienza et al., 2007; Munns and Tester, 2008). Interestingly, the cytoplasmic C-terminal of SOS1 has been suggested to function as an intracellular Na^+^ sensor (Shi et al., 2002; Qiu et al., 2002; Shabala et al., 2005), but hard evidence for this is lacking. The SOS pathway has additional components – SOS4 and SOS5 (Shi et al., 2003; Mahajan and Tuteja, 2005), which may be involved in Na^+^ and K^+^ homeostasis (Mahajan et al., 2008). The *sos4* mutants exhibit higher Na^+^/K^+^ ratio in comparison with wild-type plants (Mahajan et al., 2008). Due to the outer membrane localization, SOS5 is another potential candidate for (extracellular) Na^+^ sensing (Figure 2; Shi et al., 2003; Mahajan et al., 2008).

Thus, the SOS pathway is a key regulator of Na^+^ homeostasis, for example *via* SOS1. But, due to interaction with other regulatory proteins, it also participates in regulation of additional mechanisms of ion homeostasis: mutations in AtHKT1, which is responsible for Na^+^ translocation to the shoot (Halfter et al., 2000), suppress the sos3 mutation (Rus et al., 2001). Thus, the SOS2-SOS3 complex is involved in negative regulation of AtHKT1 during salinity stress. SOS2, in addition to modulating SOS1, can interact with vacuolar Na^+^/H^+^ exchanger (NHX) antiporters and significantly elevate their exchange activity (Zhu, 2002). SOS2 may also interact with the N-terminus of CAX1 (a H^+^/Ca^2+^ exchanger) (Qiu et al., 2002; Figure 3).

It seems likely that SOS1 activity induced by salinity not only relies on the SOS3–SOS2 complex but could be phosphorylated in a phospholipase D (PLD) signaling pathway-dependent manner (Yu et al., 2010): High Na^+^ concentrations cause an increase in enzyme activity of PLDα1 that lead to fast accumulation of phosphatidic acid (PA) as a lipid second messenger. PA in turn activates mitogen-activated protein kinase 6 (MPK6), which is capable of directly phosphorylating SOS1 (Yu et al., 2010). Loss-of-function mutants of PLDα1 and MPK6 exhibit sensitivity to salinity and accumulate more Na^+^ accumulation in the shoots.

A further SOS1 regulating mechanism originates in nuclear Ca^2+^ signaling in response to high salinity (Guan et al., 2013). Nuclear Ca^2+^ activates the Ca^2+^-binding protein RSA1, which complexes with RITF1 (RSA1 interacting transcription factor). Subsequently, the activated complex RSA1-RITF1 binds at the SOS1 promoter to augment its transcription (Figure 2; Guan et al., 2013).

Other early components in NaCl-induced sensing and signaling may be the previously mentioned ROS and cyclic nucleotides such as cGMP. Both cGMP and ROS show rapid transient increases in cytoplasmic levels after salinity stress onset (Kiegle et al., 2000; Donaldson et al., 2004). The rise of cellular cGMP can be detected within seconds after application of salinity and osmotic stress (Donaldson et al., 2004). Furthermore, cGMP inhibits Na^+^ influx in several plant species (Maathuis and Sanders, 2001; Essah et al., 2003; Rubio et al., 2003) while it can regulate transcription of various genes related to salinity stress and promote K^+^ uptake (Maathuis, 2006, 2014; Isner and Maathuis, 2016). Indeed, the work by Donaldson et al. (2004) strongly suggests cross talk between Ca^2+^ and cGMP signaling (Figure 2).

A rise in ROS is detected within minutes after the onset of salinity stress (Hong et al., 2008), and this phenomenon can activate downstream MAPK cascades (Miller et al., 2010; Maathuis, 2014). Recent studies demonstrate participation of ROS in transcriptional regulation. For example, ROS generated by plasma membrane-localized NADPH oxidase can help to stabilize AtSOS1 transcripts (Chung et al., 2008). The ROS-sensitive transcription factor ERF1 (ethylene response factor) in rice can bind to multiple promoters, including those of MAPKs, and improves general performance of plants under salinity stress (Schmidt et al., 2013). ROS are also thought to alter ion fluxes; for example, outward rectifying K^+^ channels are directly activated by ROS in Arabidopsis roots (Demidchik et al., 2010). The model further postulates that moderate K^+^ decrease in the cytosol will generate a low level of ROS designated for signaling, while high levels of salinity generate damaging ROS that activate K^+^ efflux channels and accelerate cellular K^+^ leak. Thus, fast and significant K^+^ loss could lead to acute ROS toxicity and development of programmed cell death (PCD). Indirectly, ROS such as the superoxide anion affect GORK channel transcription, providing yet another feedback loop (Tran et al., 2013).

## Ion Toxicity: How Relevant is it and Where does it Occur?

Some measure of the two main components of salt damage, osmotic stress and ion toxicity, can be obtained by comparing plants exposed to salt and those treated with equiosmolar quantities of an inert osmoticum such as polyethylene glycol (PEG). Many of such studies are available, and the majority shows overwhelmingly that the osmotic stress component makes up a much larger fraction than the ion toxicity component (Castillo et al., 2007; Zhao et al., 2010).

Salt toxicity directly relates to ion concentrations and can manifest itself in all cell compartments though is usually assumed to be associated with the cytoplast. Thus, reliable measurements of cellular Na^+^ and Cl^−^ concentrations are crucial for proper evaluation of toxicity but such measurements have only been conducted for relatively few plants. Values for [Na^+^]_vac_ [Cl^−^]_vac_ and [K^+^]_vac_ using X-ray analyses (Chen et al., 2014), microelectrodes (Carden et al., 2003), dyes (Wissing and Smith, 2000; Wu et al., 2015a), and whole tissue extraction (Patishtan et al., 2018) show that both [Na^+^]_vac_ and [Cl^−^]_vac_ vary widely from tens of mM to 1 M in many halophytes (Zhao et al., 2005; Flowers and Colmer, 2008).

Obtaining truthful values of cytoplasmic ion concentrations is fraught with difficulty because of the small size of this compartment (see Kronzucker and Britto, 2011; Flowers et al., 2014) for a discussion of methodological aspects). Though all approaches generate some artifacts, techniques based on the use of ratiometric fluorescent dyes such as SBFI and ion selective electrodes (Carden et al., 2003) are preferable, since they record in real time and do not require any tissue preparation. Data for [Na^+^]_cyt_ determined with SBFI (Halperin and Lynch, 2003; Kader and Lindbergh, 2005) vary between ~5 and 70 mM with external NaCl concentrations between 5 and 100 mM. Data obtained with electrodes (Carden et al., 2003) fall in the range 5–25 mM when barley was exposed to 200 mM NaCl. Data for cytoplasmic [K^+^] are less variable and approximately between 70 and 90 mM (Walker et al., 1996; Carden et al., 2003). To measure cytoplasmic Cl^−^, genetic GFP-based reporters such as Clomeleon (Markova et al., 2008) and fluorescent dyes have been exploited in animal cells showing values typically between 50 and 80 mM (Salomonsson et al., 1993). Unfortunately, these techniques have not (yet) been used to determine [Cl^−^]_cyt_ in plant cells. Gerson and Poole (1972) used microelectrodes to measure [Cl^−^]_cyt_ in nonvacuolate root tip cells and found ~30 mM in the presence of 60 mM KCl (comparable to 60 mM NaCl). Multiple studies from compartmental analysis and X-ray studies (White and Broadley, 2001; Flowers et al., 2014) show values ranging from ~45 to 140 mM in the presence of 50–100 mM NaCl. In all, these numbers suggest that during moderate salinity (~5–10 dS m^−1^), the maximum [Na^+^]_cyt_ (60–70 mM) is similar to [K^+^]_cyt_ (~70–90 mM) and cytoplasmic K:Na ratios are unlikely to drop far below unity. Data for [Cl^−^]_cyt_ are more scarce but suggest a comparable or slightly higher range to that of Na^+^ and K^+^.

Data for the apoplast compartment vary greatly. Work with pea and spinach showed a substantial difference between these species (Speer and Kaiser, 1991). Pea apoplasts reached concentrations of around 90 and 200 mM for Na^+^ and Cl^−^, respectively, while corresponding levels in spinach did not exceed 10 and 15 mM. Comparative studies with canola and rice (Gao et al., 2016) determined apoplastic Na^+^ levels of ~130 (canola) and ~100 (rice) mM after 20 day treatment with 150 (canola) and 100 (rice) mM NaCl. Salinization of *Vicia faba* beans with 50, 75, or 100 mM NaCl caused apoplastic Na^+^ to rise to ~5, 30, and 100 mM (Shahzad et al., 2013).

The presence of high concentrations of Na^+^ and Cl^−^ can disturb water structure *via* kosmo- and chaotropic effects, inhibit enzymes, and create nutritional imbalance. The higher charge density of Na^+^ compared to K^+^ means it behaves as a weak “kosmotrope” that organizes and immobilizes water structure around itself. Kosmotropy affects hydrogen bonding between water molecules and polar groups of proteins and nucleic acids, potentially interfering with their biochemical activity. K^+^ has a less tight hydration shell thus behaving as a weak “chaotrope.” A discernible kosmotropic effect of Na^+^ typically requires concentrations of >200 mM. Furthermore, molecular dynamic simulation shows that Na^+^ and K^+^ influence protein or DNA in a similar manner (Cheng et al., 2006). In fact, the tropic effects of anions are typically greater than those of cations but Cl^−^ (like K^+^) is a very weak kosmotrope. Thus, in the presence of moderate salinity and the prevailing cytoplasmic concentrations, the potential impact of K^+^, Na^+^, and Cl^−^ on solvent properties would be negligible.

Another potential ion toxicity mechanism that is often referred to in the context of salinity stress is the requirement of many enzymes to bind K^+^ which can be disrupted by Na^+^ displacing K^+^. What is striking is that in many cases the older literature reports almost identical effects of K^+^ and Na^+^ on enzyme and polysome activity (Johnson et al., 1968; Greenway and Osmond, 1972; Osmond and Greenway, 1972; Brady et al., 1984). Enzymes, such as starch synthase and glucose-6-phosphate dehydrogenase (Johnson et al., 1968), show maximum activity in the presence of 50–100 mM monovalents but do so irrespective of it being K^+^ or Na^+^. A recent report on the kinetic properties of rice pyrroline-5-carboxylate reductase agrees with this notion (Forlani et al., 2015). These findings indicate that Na^+^ can substitute K^+^ without significant problems for many biochemical activities. However, in other cases, the requirement for K^+^ is more specific; for example, the Kcat for Na^+^ activation of pyruvate kinase, a classical example of a K^+^ stimulated enzyme (Kachmar and Boyer, 1953), is only about 8% compared to that for K^+^. Nevertheless, the approximately 10-fold higher affinity of this enzyme for K^+^ (Kachmar and Boyer, 1953) compared to Na^+^ ensures that a Na:K ratio of more than 3 would be needed to significantly reduce enzyme activity. Similarly, studies on other enzymes (Shelp and Atkins, 1983; Gibrat et al., 1990) show Km values for K^+^ that are often very low (10–15 mM) while those for Na^+^ are much higher (>100 mM). In a few studies, the effect of anions was scrutinized and, as far as is known, in general Cl^−^ has no detrimental effect on enzyme activity at [Cl^−^]_cyt_ below ~80–100 mM (Greenway and Osmond, 1972; Brady et al., 1984), which is also implied by routine measurement of 60–90 mM [Cl^−^]_cyt_ in animal cells.

In contrast to cytoplasmic concentrations, vacuolar levels of Na^+^ and Cl^−^ readily reach several hundred mM. For example, a recent study on rice cultivars exposed to 50 mM NaCl showed tissue [Na^+^] of up to 600 mM (Patishtan et al., 2018). Consequently, vacuolar Na/K ratios can easily exceed values of 3 or 4. The central vacuole plays a role in ionic homeostasis, pH regulation, and osmotic adjustment. As lytic compartment, the vacuole contains hydrolases, phosphatases, and phosphoesterases (Boller and Kende, 1979) and thus is linked to protein turnover processes like ubiquitination. In spite of extensive information regarding the vacuolar proteome, to our knowledge no vacuole-specific enzymes have been tested for the functional implications of high Na^+^ and Cl^−^ concentrations. The above data suggest that when glycophytes are exposed to moderate salinity stress (~50–150 mM) ion toxicity in the cytosol is unlikely to be problematic but, in contrast, whether vacuolar enzymes can maintain functionality when typically surrounded by much higher salt concentrations is a question that needs to be answered urgently.

In summary, in spite of considerable inward gradients for Na^+^, most data suggest that plants adequately prevent accumulation of Na^+^ and Cl^−^ in the cytosol beyond 50–80 mM, even in the presence of 100–150 mM external Na^+^. Apoplastic levels are in the same order of magnitude. Consequently, if ion toxicity occurs in these compartments, it is probably of limited magnitude. The situation in the vacuole where ion concentrations can easily exceed 500 mM may be quite different. Alternatively, the ionic component of salinity stress could manifest itself *via* generic parameters such as the membrane potential (e.g. Gill et al., 2017) rather than specific ionic interactions. Since many transport processes are voltage dependent, depolarization could influence a plethora of processes including the uptake of essential nutrients.

## What is the Role of Potassium in Salinity Stress?

K^+^ is the most abundant cation in plant cells and an essential nutrient that is important for many enzymatic reactions, ionic and pH homeostasis and maintaining adequate membrane potential (Maathuis 2009; Ahmad and Maathuis, 2014). Cytosolic K^+^ is also an important determinant of plant adaptive responses to a broad range of environmental stresses (e.g. Shabala and Pottosin, 2014). In hydrated form, Na^+^ and K^+^ are chemically and structurally very similar and some biophysical roles of K^+^, particularly generating turgor, can be fulfilled by Na^+^. Nevertheless, K^+^ is uniquely required for many physiological and biochemical processes, whereas Na^+^ is not. The transport systems involved in the uptake and distribution of K^+^ and Na^+^ in combination are key determinants of plant salinity tolerance due to their ability to determine tissue and cytosolic K^+^/Na^+^ ratios, parameters that are generally believed to impact greatly on salt tolerance (Maathuis and Amtmann, 1999; Shabala and Cuin, 2008). Influx of Na^+^ worsens the K/Na ratio, and this is further exacerbated by salt stress-induced K^+^ loss, a phenomenon that is often more pronounced in salt-sensitive species (Chen et al., 2007; Wu et al., 2018). GORK (guard cell outward rectifying K^+^ channel) type and ROS-activated NSCC-type channels are likely to mediate the main fraction of K^+^ efflux (Jayakannan et al., 2013; Wu et al., 2015b). In addition, salt stress-induced K^+^ leakage can cause PCD (Figure 2; Demidchik et al., 2014).

Interaction between K^+^ and Na^+^ transport has been described in many studies and many forms. For example, salinity affects K^+^ transporter transcription as exemplified by work on OsAKT1 (an inward rectifying K^+^ channel) where *in situ* hybridization showed that OsAKT1 transcription is downregulated after exposure to salinity in a cell type and cultivar-specific manner (Golldack et al., 2003). Other studies have shown upregulation of the phloem localized AKT2/3 and stelar root tissue located SKOR (an outward rectifying K^+^ channel) by salinity (Marten et al., 1999; Maathuis, 2006). These data in combination suggest that salinity may increase K^+^ circulation *via* the vascular bundles (Maathuis, 2006; Shabala and Cuin, 2008). Endomembrane channels such as the vacuolar TPKs (two pore K^+^ channels) are also likely to play an important role. The expression of tobacco TPK1a was increased around twofold by salt stress or osmotic shock (Hamamoto et al., 2008), whereas TPK overexpression in tobacco cells increased their resistance to salt stress (Wang et al., 2013). Post-translational modulation of TPK1 also impacts on salt tolerance as was shown for AtTPK1, which becomes phosphorylated by a Ca^2+^-dependent kinase (CDPK3; Latz et al., 2013) (Figure 2).

It is generally assumed that increased levels of K^+^ mitigate against salt stress, but this may be an oversimplification. Indeed, recent experimental data suggest that Na^+^ toxicity and water deficit are often not the key causes of plant growth inhibition by NaCl in Arabidopsis (Álvarez-Aragón et al., 2016). Rather, the overaccumulation of Na^+^ plus K^+^ might trigger growth reduction in NaCl-treated Arabidopsis plants, for example, *via* effects on stomatal regulation or systemic stress responses that lower growth (Álvarez-Aragón et al., 2016).

These salinity-induced effects on K^+^ transport are often variety, tissue and cell specific. For example, salt-tolerant barley varieties are capable to better retain K^+^ in the roots in comparison with sensitive genotypes (Chen et al., 2007), whereas different Arabidopsis ecotypes show widely varying K^+^/Na^+^ ratios (Álvarez-Aragón et al., 2016). Salt-induced K^+^ efflux in wheat and barley mesophyll cells of salt-sensitive varieties is significantly higher than that in salt-tolerant varieties (Wu et al., 2013, 2015c), and in contrast to cultivated barley, halophytic wild barley relatives exhibited much better tissue K^+^ retention (Garthwaite et al., 2005). At the tissue level, the higher salt sensitivity of barley root apex in comparison with the mature root zone was explained by a much greater NaCl-induced K^+^ efflux in root apex (Shabala et al., 2006). In turn such observations may be caused by the cell and tissue specificity of K^+^ transporters. In the root, mature zone efflux is mediated primarily by GORK-type channels (Chen et al., 2007; Shabala et al., 2007a; Wu et al., 2013; Chakraborty et al., 2016), whereas in the elongation zone NSCC channels fulfill this function (Bose et al., 2014). These findings suggest that the ability of plants to retain K^+^ in various tissues is an important feature of plant salt tolerance (Wu et al., 2018).

Multiple reports suggest that K^+^ could play important role in cell signaling during salinity (Shabala 2009, 2017; Anschütz et al., 2014; Shabala and Pottosin, 2014; Wu et al., 2018). In this context, K^+^ can cause cell- and tissue-specific metabolic changes and drive a “metabolic switch” to inhibit energy-dependent biosynthetic processes. The ensuing reduction or arrest of plant growth saves energy, which in turn augments the capacity for the synthesis of compounds that help in defence and repair of cellular systems (Demidchik et al., 2014). A K^+^-signaling function can also be envisaged in its capacity to generate PCD (Huh et al., 2002; Shabala, 2009). The physiological role of PCD during salinity is still discussed, but many experimental data directly link salinity stress, K^+^ leak, and PCD. For example, Arabidopsis mutants with loss of function in GORK channels exhibit slower development of salinity-related PCD (Demidchik et al., 2010), an observation that can be mimicked with channel blockers such as TEA (Osakabe et al., 2013). Interestingly, overexpression of the animal antiapoptotic CED-9 gene in tobacco mesophyll cells led to a reduction of stress-induced K^+^ efflux and improved plant salt tolerance (Shabala et al., 2007b). Thus, stress-induced K^+^ leakage, ROS, and PCD are likely to be tightly connected (Demidchik et al., 2010).

In all, K^+^ homeostasis is intricately linked to salt tolerance. At its most basic level, this involves substitution of K^+^ in its biochemical roles, but more complex relationships may exist such as K^+^-related PCD and the role of K^+^ as a signaling moiety that modulates metabolic pathways. Where the latter processes are concerned, more detailed studies at the molecular level are needed for example on the exact mechanism and cytosolic K^+^ concentrations that cause PCD.

## Conclusions

The ever-increasing salinization of arable land will require multipronged solutions of which crops with increased tolerance is one. Exploiting genetic diversity will help achieving this objective but would be far more effective when combined with a comprehensive understanding of the molecular tolerance mechanisms. Great progress has been made in the last decades but yet many of the basic processes that contribute to tolerance are only partially understood. Further studies are urgently needed to unravel the details of Na^+^, and especially Cl^−^, uptake mechanisms. Mapping of toxicity at the cell and tissue level will aid in setting targets for tolerance improvement. Perception, sensing, and signaling chains lack important components in particular those at the beginning of pathways, whereas greater understanding of the role of other minerals such as K^+^ should enable us to mitigate salt stress by manipulating uptake and distribution of these nutrients.

## Author Contributions

All authors listed have made a substantial, direct and intellectual contribution to the work, and approved it for publication.

### Conflict of Interest Statement

The authors declare that the research was conducted in the absence of any commercial or financial relationships that could be construed as a potential conflict of interest.
